# Supplementary material for: Memoryless drop breakup in turbulence
Source: Sci Adv. 2022 Dec 16;8(50):eabp9561. doi: 10.1126/sciadv.abp9561 (PMC9757738; doi:10.1126/sciadv.abp9561)
Supplement: 20221216-1 [file sciadv.abp9561.v1.pdf]

CORRECTION POSTED 27 MARCH 2023

PHYSICAL SCIENCES

Copyright © 2022 The Authors, some rights reserved; exclusive licensee American Association for the Advancement of Science. No claim to original U.S. Government Works. Distributed under a Creative Commons Attribution License 4.0 (CC BY).

Memoryless drop breakup in turbulence

Alberto Vela-Martín[1]*† and Marc Avila[1,2]*†

The breakup of drops and bubbles in turbulent fluids is a key mechanism in many environmental and engineering processes. Even in the well-studied dilute case, quantitative descriptions of drop fragmentation remain elusive, and empirical models continue to proliferate. We here investigate drop breakup by leveraging a novel computer code, which enables the generation of ensembles of experiments with thousands of independent, fully resolved simulations. We show that in homogeneous isotropic turbulence breakup is a memoryless process whose rate depends only on the Weber number. A simple model based on the computed breakup rates can accurately predict experimental measurements and demonstrates that dilute emulsions evolve through a continuous fragmentation process with exponentially increasing time scales. Our results suggest a nonvanishing breakup rate below the critical Kolmogorov-Hinze diameter, challenging the current paradigm of inertial drop fragmentation.

INTRODUCTION

The dynamics of drops and bubbles in turbulence plays an important role in the emulsification of immiscible liquids in food industries (*1*, *2*), sprays and atomization in combustion (*3*, *4*), rain formation in clouds (*5*), rainfall (*6*), and the liquid-gas exchange in the oceans (*7*–*9*). In these examples, the disperse phase breaks down into smaller fluid particles due to the ambient turbulence, but coalescence can increase their size. The competition between these two processes controls the particle size distributions, which determine important properties of immiscible mixtures such as the interfacial area.

The theoretical foundations for the breakup of fluid particles larger than the viscous scale in turbulence ($\eta = \nu^{3/4}/\varepsilon^{1/4}$, where ε is the kinetic energy dissipation and ν the kinematic viscosity of the carrier fluid) were laid out by Kolmogorov (*10*) and Hinze (*11*). They assumed that a particle of diameter d is deformed by turbulent velocity fluctuations up to the same scale, $\overline{\Delta u^2(d)} \approx (\varepsilon d)^{2/3}$, and postulated that breakup would occur when the kinetic energy of these fluctuations exceeded the interfacial energy. This occurs when the dimensionless Weber number ($\mathrm{We} = \rho\, \varepsilon^{2/3} d^{5/3}/\sigma$, where ρ is the density of the carrier phase and σ is the surface tension) exceeds a critical value of order unity (*10*, *11*). Accordingly, breakup is inhibited when the diameter falls below the corresponding critical (Kolmogorov-Hinze) diameter

$$d_{\mathrm{KH}} = C\left(\frac{\rho}{\sigma}\right)^{-3/5} \varepsilon^{-2/5} \tag{1}$$

where C is a constant. Hinze (*11*) fitted this equation to experimental measurements of d_{95}, the diameter below which 95% of the volume of the disperse phase is contained, and obtained good agreement for $C = 0.725$. Although deviations from this value have been reported in the literature (*12*–*15*), the Kolmogorov-Hinze diameter d_{KH} is extremely useful because it can be used to estimate d_{95} from material properties and the dissipation only. The latter can be estimated from the power input or from standard single-phase computational fluid dynamics simulations of the experimental device (*12*).

Despite its remarkable success and widespread use, the approach pioneered by Kolmogorov and Hinze does not provide information on the time scale of the fragmentation process, which is essential to predict the evolution of the particle size distributions. In sprays or in emulsions, it is very difficult to track individual drops, and extracting breakup rates from the evolution of the size distributions is extremely challenging (*16*). Hence, there have been a number of experimental studies in which single drops (or bubbles) are injected and tracked as they pass a turbulent flow region (*3*, *17*–*23*). In such experiments, fluid particles are deformed by turbulence and may break before exiting the turbulent region. However, there is no commonly accepted method to determine breakup rates from the experimental data, and the results disagree quantitatively (*24*). Possible sources of discrepancies are different flow setups and observation times, insufficient statistics, or the methodology to extract the breakup rate. In a nutshell, the dynamics of breakup remains poorly understood (*2*), and phenomenological models continue to proliferate in the literature (*15*, *18*, *25*–*29*). In this work, we show that drop breakup in homogeneous isotropic turbulence obeys a memoryless process whose rate depends only on the Weber number. We determined the scaling of the breakup rate and obtained simple, accurate predictions of the evolution of drop size distributions in the dilute regime.

RESULTS

We leveraged our novel pseudo-spectral Graphich Processing Unit (GPU) code described and validated in (*30*) and (*31*) to perform more than 30,000 independent simulations of drop breakup at several We and Reynolds numbers, $\mathrm{Re}_\lambda = \lambda u'/\nu$, where $\lambda = \sqrt{15(\varepsilon/\nu)}u'$ is the Taylor microscale, and u' is the root mean square of the velocity fluctuations (see Table 1 for a summary of all simulations). The procedure for each simulation was as follows. A single spherical drop of diameter $d \gg \eta$ was placed in a turbulent carrier fluid and was let to evolve. The drop deformed, while visiting regions of intense turbulence, and relaxed back toward a sphere in regions of quiescent motion. Eventually, it broke down into two or

[1]Center of Applied Space Technology and Microgravity (ZARM), University of Bremen, Bremen 28359, Germany. [2]MAPEX Center for Materials and Processes, University of Bremen, Bremen 28359, Germany.
*Corresponding author. Email: alberto.vela.martin@zarm.uni-bremen.de (A.V.-M.); marc.avila@zarm.uni-bremen.de (M.A.)
†These authors contributed equally to this work.

Table 1. Number of independent simulations for each Reynolds and Weber number at high ($\mathcal{N}_{HR}$) and low ($\mathcal{N}_{LR}$) resolution. In addition, we ran 257 high-resolution simulations at $Re_\lambda = 96$ and viscosity ratio $\gamma = 2$ and 215 simulations at the same Re_λ and $\gamma = 0.5$. Last, we also ran 200 high-resolution simulations at $Re_\lambda = 58$ and We = 2.18 with initially ellipsoidal drops (see section S3).

	$Re_\lambda = 31$		$Re_\lambda = 58$		$Re_\lambda = 96$	
**We**	$\mathcal{N}_{LR}$	$\mathcal{N}_{HR}$	$\mathcal{N}_{LR}$	$\mathcal{N}_{HR}$	$\mathcal{N}_{LR}$	$\mathcal{N}_{HR}$
1.45	–	–	1687	224	–	–
1.82	1208	–	1475	342	–	180
2.18	1208	–	2001	201	–	245
2.55	1208	–	1834	201	–	248
2.91	1208	–	3477	216	–	248
3.27	1208	–	3217	216	–	486
3.63	1208	–	3501	248	–	488
4.36	2008	–	2008	248	–	328
4.72	1789	–	2008	176	–	488
5.45	–	–	–	248	–	488
6.18	–	–	–	205	–	–
7.27	–	–	–	255	–	–

more drops, as exemplified in Fig. 1. To ensure well-converged statistics, each run was initialized with a different (decorrelated) turbulent flow field, and many hundreds of runs were performed for each (We, Re_λ) pair in the ranges We ∈ [1.45,7.27] and Re_λ ∈ [31,96]. For the highest Reynolds number, $Re_\lambda = 96$, the diameter of the drop lies within an incipient inertial range of scales (see section S1). The breakup time, rendered dimensionless with the inertial time scale of the drop, $t_d = (d^2/\varepsilon)^{1/3}$, and the daughter size distribution were stored for each simulation. We first considered two inmiscible fluids of equal density and viscosity and subsequently varied the ratio $\gamma = \nu_d/\nu$, where ν_d is the kinematic viscosity of the drop.

We let $P(t)$ denote the probability that a drop breaks before time t. Then, $1 - P(t)$ is the probability that a drop survives beyond time t. As shown in Fig. 2A, $1 - P(t) = \exp[-(t - t_0)\kappa(\mathrm{We})]$, where t is the observation time, t_0 is an equilibration time, and κ is the dimensionless breakup rate. The equilibration time includes the time for the drop to settle into the flow conditions, and the minimum time needed for breakup when the drop has reached a critically deformed state (*32*). Beyond the equilibration time, distributions are exponential and depend only on the breakup rate, κ, reflecting that the probability of breakup is constant and therefore independent of how long the drop has been exposed to turbulent fluctuations (see fig. S2). Hence, drop breakup is a "memoryless" process, statistically similar to the decay of radioactive materials or the decay (*33*) and splitting (*34*) of turbulent puffs in pipe flow. Exponential distributions of drop and bubble breakup times are visible in previous experimental (*35*) and numerical (*36*) data but are rationalized and fully characterized here. To further test the memoryless nature of breakup, we checked that the breakup rate does not change when the initial drop is not spherical but ellipsoidal (see section S3). We note that for high Weber numbers, We $\gtrsim$ 3, most breakups occur well before the equilibration time. This regime is characterized by the rapid, violent deformation of the interface at several

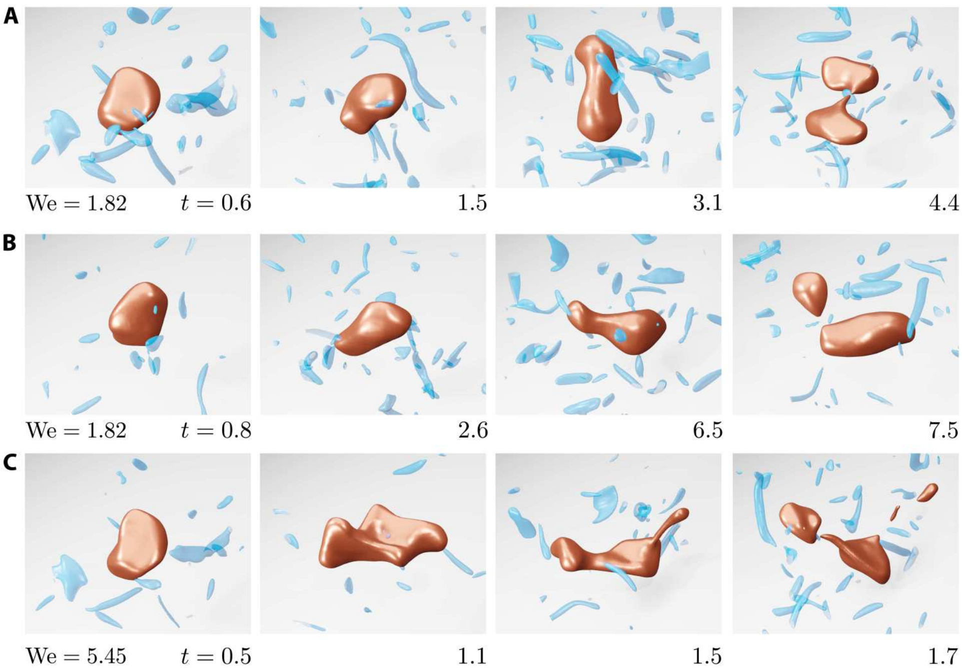

Fig. 1. Drop deformation and breakup in homogeneous isotropic turbulence. Temporal evolution of the drop-breakup process for three independent simulations at We = 1.82 (**A** and **B**) and We = 5.45 (**C**). The Reynolds number is $Re_\lambda = 58$, and times are made dimensionless with the inertial time scale of the drop, $t_d = (d^2/\varepsilon)^{1/3}$. At $t = 0$, a spherical drop is placed into the turbulent flow. The drop interface is shown as red, whereas isosurfaces of enstrophy $5\langle\Omega\rangle$, where $\Omega = |\boldsymbol{\omega}|^2$, and $\boldsymbol{\omega} = \nabla \times \mathbf{u}$ is the vorticity vector, are shown in blue. For clarity, the snapshots were produced in a frame moving with the center of mass of the drop.

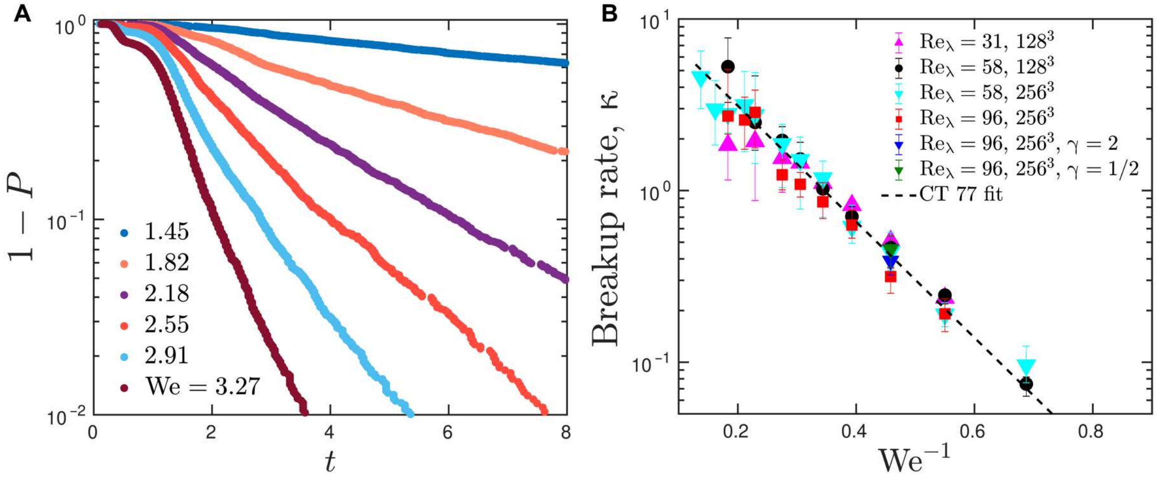

Fig. 2. Drop breakup is a memoryless process with exponentially increasing breakup times as surface tension increases. (**A**) Probability distributions of drop survival for We as indicated in the legend. P is the probability that a drop breaks before time t. Hence, the plotted quantity, $1-P$, is the probability that a drop survives up to time t. The distributions were obtained from all first breakup times in ensembles of simulations at $\mathrm{Re}_\lambda = 58$ and $N = 128^3$. All distributions are of the form $\exp[-(t-t_0)\kappa(\mathrm{We})]$, where $t_0 \approx 2$ is an equilibration time. The exponential form of the distributions indicates that breakup is a memoryless process with dimensionless breakup rate κ (and mean time $\tau = \kappa^{-1}$). (**B**) Breakup rate as a function of the inverse of the Weber number We^{-1}. Symbols denote simulations at different Reynolds numbers, Re_λ, numerical resolutions, N^3, and viscosity ratios, γ. The dashed line depicts the fit to a model proposed by Coulaloglou and Tavlarides (*25*) (CT 77), $\kappa = c_1 \exp[-2c_2/\mathrm{We}]$, where the dimensionless constants $c_1 = 14.8$ and $c_2 = 3.9$ are obtained from a least-square fit to the data.

length scales ℓ, with $\eta \le \ell \le d$, and the formation of many daughter drops, as shown in Fig. 1C.

The dimensionless breakup rates computed with a maximum likelihood estimator (see section S2) are shown in Fig. 2B and are well approximated by the equation

$$\kappa = c_1 \exp\left(-2c_2 \mathrm{We}^{-1}\right) \tag{2}$$

Specifically, the breakup rate depends neither on the Reynolds number of the flow nor on the viscosity ratio, γ, and is uniquely determined by the Weber number. In contrast to the original hypothesis of Kolmogorov (*10*) and Hinze (*11*), our results suggest that there is no critical We for drop breakup. In the limit of strong surface tension, We → 0, the characteristic breakup time, $\tau = \kappa^{-1}$, rises exponentially, but the scaling implies a finite breakup time for all We > 0. This is similar to the Reynolds number scaling of the decay (*33*) and splitting (*34*) times of turbulent puffs in pipe flow. An exponential dependence of the breakup rate on We^{-1} was first proposed by Coulaloglou and Tavlarides (*25*) in the context of phenomenological models of emulsion dynamics. Because of the memoryless property unveiled here, κ completely describes the breakup probability of single drops and the breakup rate in emulsions and, together with the daughter-drop statistics shown in fig. S4A, can be directly implemented in population-balance models (*37*).

In the range of We investigated in this work, breakup can be assumed binary for practical purposes (see fig. S4A). This observation, together with the memoryless nature of breakup, enables a simple stochastic model of the asymptotic evolution of dilute

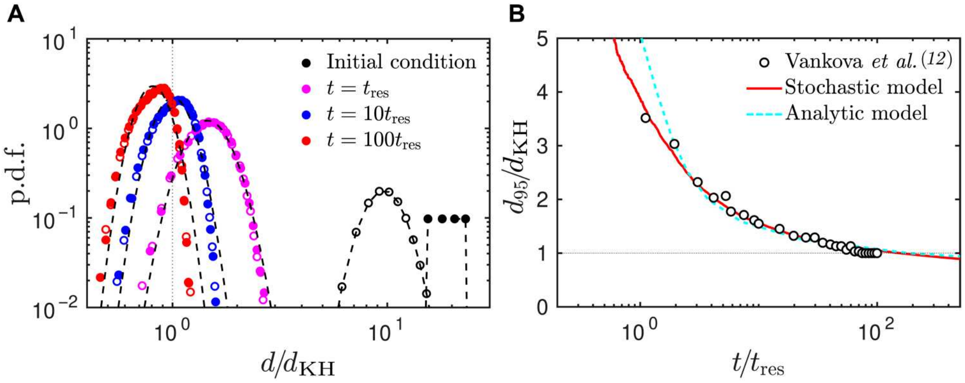

Fig. 3. Dilute emulsions evolve through a fragmentation process with exponentially increasing time scales. (**A**) Temporal evolution of the drop diameter probability density function (p.d.f.) in the stochastic breakup model for two different initial conditions; empty markers correspond to a log-normal distribution with average $10d_{\mathrm{KH}}$ and solid markers to a uniform distribution with average $20d_{\mathrm{KH}}$. The dashed lines show least-square fits to the drop-size distributions with a lognormal. (**B**) Temporal evolution of d_{95} in the stochastic breakup model (solid line) and of the mean diameter in the analytic model (dashed line; eq. S7) compared to experimental measurements of Vankova *et al.* (*12*). Here, t_{res} is the characteristic residence time of the drops in the turbulent region where breakup can occur in each passage through the emulsifier (see eq. S6). To define d_{KH}, we used $C = 0.86$, as in Vankova *et al.* (*12*).

emulsions. Starting from an initial drop with diameter d_0 (or Weber number We_0), the dimensional breakup time is computed according to a memoryless process, $t_{b_0} = -t_{d_0} \log X/\kappa(We_0)$, where $X \in [0,1]$ is a uniform random variable. The diameter of the largest daughter is randomly extracted from the distribution shown in fig. S4A, which approximates well the results from our direct numerical simulations. The diameter of the second daughter follows from mass conservation. The same procedure is applied to the two daughters [and so forth in a cascade fashion, similar to (*38*)], until a desired time, and is repeated for a large number of initial drops to obtain a temporally resolved drop size distribution. As shown in Fig. 3A, our stochastic model predicts that the drop size distribution quickly loses memory of the initial distribution and converges toward a function uniquely determined by the constants c_1 and c_2 in Eq. 2, and the distribution of daughter drops. This universality of the fragmentation process, further demonstrated infig. S4B, is a direct consequence of the memoryless nature of drop breakup and, to a lesser extent, of the random nature of the daughter sizes.

The evolution of $d_{95}(t)$ was extracted from the data of our model in Fig. 3A and is compared in Fig. 3B to the experimental measurements of Vankova *et al.* (*12*), who repeatedly passed an emulsion through the same device (see section S4 for details of the comparison). Our model prediction is in excellent agreement with their measurements and shows that the emulsification process slows down markedly as $d_{95}(t) \approx d_{KH} \approx 9$ μm, reflecting the exponential decrease of the breakup rate in Eq. 2. Vankova *et al.* (*12*) reported a steady state after 100 passages through the emulsifier, whereas Eq. 2 suggests that the fragmentation process should continue. However, it would take about 1000 passages to further reduce d_{95} by 15% only, provided that coalescence could still be neglected. In section S5, we derive an analytical prediction of the time necessary to reach a desired mean diameter in a dilute emulsion (eq. S7). As shown in Fig. 3B, our prediction is in good agreement with the experiments (*12*).

DISCUSSION

We have shown that drop breakup in isotropic turbulence is described by a memoryless process whose rate depends only on the Weber number. These results provide a direct quantification of the time scales that govern the evolution of dilute turbulent emulsions. In particular, simple stochastic and analytical fragmentation models derived from our data accurately reproduce the evolution of the drop diameter in experiments, showing that dilute emulsions evolve through a continuous fragmentation process with exponentially increasing time scales.

The scaling of the breakup rate obtained here is consistent with a nonvanishing breakup probability below the critical Kolmogorov-Hinze diameter (*10*, *11*). This challenges the established paradigm of drop breakup and suggests that fragmentation processes may equilibrate at smaller diameters than previously thought, albeit in time scales that have not been previously reached in experiments or simulations. We argue that drop breakup below the Kolmogorov-Hinze scale is a natural consequence of the fluctuating nature of turbulent flows and would occur because of the interaction of drops with the most intense eddies. In particular, the breakup of very small drops and bubbles should be possible due to the increasing frequency of very intense and extreme events (eddies) with the Reynolds number, as a result of intermittency (*39*–*41*). The scaling of the breakup rate revealed here suggests a connection between drop breakup and extreme events in turbulence, where the exponential dependence with the Weber number would emerge from the limit Gumbel distribution, as conjectured for the decay and splitting of turbulent puffs in pipe flow (*42*–*44*), and the time scales of breakup would be determined by the memoryless waiting times between intense events in turbulence (*45*, *46*). This connection could be exploited to develop breakup models, which could be tested in simulations and experiments at higher Reynolds numbers.

A key aspect of this work has been to characterize the dynamics of single drops with large ensembles of independent, fully resolved simulations. This approach can be readily extended to fluid pairs with vastly different densities and viscosities, such as drops in air or bubbles in liquids, and to include the effect of surfactants (*47*). Last, by considering two fluid particles immersed in turbulence, their coalescence could also be investigated with ensembles of simulations. The resulting statistical description of the coalescence process, together with the one provided here for breakup, would pave the way for accurate population-balance (*37*) or breakup-cascade (*38*) models beyond the dilute regime and would advance our understanding of many processes involving drops and bubbles in turbulent environments (*2*, *4*–*6*, *8*). We anticipate that the ratio of breakup to coalescence rates may determine the stationary particle size distribution, in a similar manner as the ratio of decay to splitting rates determines the laminar-turbulent patterns in pipe (*34*) and Couette (*48*) flows.

MATERIALS AND METHODS

Governing equations

We consider the incompressible Navier-Stokes equations coupled to the Cahn-Hilliard equations (*49*)

$$\begin{aligned} \partial_t u_i + u_j \partial_j u_i &= -\frac{1}{\rho}\partial_i p + 2\partial_j \nu_c S_{ij} + f_i - \frac{1}{\rho} c \partial_i \phi, \\ \partial_i u_i &= 0, \\ \partial_t c + u_j \partial_j c &= \varkappa \partial_{kk} \phi \end{aligned} \tag{3}$$

which describe the evolution of an immiscible binary mixture of fluids with equal density, ρ, and different kinematic viscosity. Here, u_i is the ith component of the velocity vector, p is the generalized pressure, $S_{ij} = 1/2(\partial_i u_j + \partial_j u_i)$ is the rate-of-strain tensor, and f_i is a body-force term per unit mass. Repeated indices imply summation. The concentration of each component in the mixture is represented by a phase field $c \in [-1,1]$, where $c = \pm 1$ are the pure components, with $c = -1$ representing the carrier, and $\varkappa$ is the mobility, which determines the relaxation time of the fluid-fluid interface. The kinematic viscosity depends linearly on the concentration $\nu_c = \nu + (c + 1)\nu_d/2$, where ν is the kinematic viscosity of the carrier and ν_d that of the drop. The immiscibility is modeled through a chemical potential

$$\phi = \beta(c^2 - 1)c - \alpha \partial_{kk} c \tag{4}$$

where α and β are model parameters that fix the width of the interface between the components, $\varepsilon = 4\sqrt{2\alpha/\beta}$, and the surface tension

(*49*)

$$\sigma = \frac{4}{3}\sqrt{\frac{\alpha\beta}{2}} \quad (5)$$

In the limit of vanishingly small interface width, $\epsilon \to 0$, the classical stress balance at fluid-fluid interfaces (i.e., the sharp-interface limit) is recovered (*50*). The model parameters $\varkappa$, α, and β form two-dimensional numbers, the Cahn and Peclet numbers

$$\mathrm{Cn} = \sqrt{\frac{\alpha}{\beta d^2}}, \mathrm{Pe} = \frac{u' d^2}{\varkappa\sqrt{\alpha\beta}} \quad (6)$$

which determine the dimensionless interface thickness and the relaxation time of the interface, respectively. Magaletti *et al.* (*50*) have shown that $\mathrm{Pe} \propto \mathrm{Cn}^{-2}$ is required to ensure a consistent physical behavior of the Cahn-Hilliard–Navier-Stokes equations (CHNS) (Eq. 3). Following their recommendation we set $\mathrm{Pe} = (3Cn^2)^{-1}$. For a review of the CHNS phase-field method and a comparison with other methods, see (*51*).

Numerical method

We integrate Eq. 3 in a triply periodic domain of size L by projecting the equations on a basis of $N/2$ Fourier modes in each direction. To sustain turbulence in a steady state, we implement a linear body force, $\hat{f}_i = \zeta\hat{u}_i$, that is only applied to wave numbers $k < 2$, where $\hat{\cdot}$ denotes the Fourier transform, and k is the wave number magnitude. The forcing coefficient ζ is set so that, at each time, the total kinetic energy per unit time injected in the system is constant, and its value is set to keep a desired numerical resolution, $k_{\max}\eta$, where $k_{\max}$ is the maximum wave number. This forcing leads to temporal fluctuations of the volume-average dissipation. The SD of these fluctuations is of the order of 10% its average for all Reynolds numbers. Moreover, the local dissipation in the carrier fluid, $2\nu S_{ij}S_{ij}$, fluctuates strongly with respect to the mean (*39*). We have performed simulations in a mesh with low resolution (LR), $N = 128$, and a mesh with high resolution (HR), $N = 256$. Further details of the code and the simulations are presented in (*31*).

The numerical simulations reproduce the evolution of a single drop of diameter $d = 0.3L$ in isotropic turbulence. For the three Reynolds numbers considered here, $Re_\lambda = 31$, 58, and 96, the drop diameter in Kolmogorov units is $d/\eta = 22$, 44, and 89, respectively. For these drop diameters, breakup is not affected by the linear forcing of the flow, and the drop does not modify the structure of the surrounding turbulence, as shown in detail in (*31*). Thus, these simulations reproduce the evolution of drops in a dilute emulsion, where drops neither interact with each other nor change the structure of the flow.

Ensembles of single-drop simulations

Our research database comprises ensembles of many independent single-drop breakup simulations, which were generated following the same procedure. Each run starts with a different initial turbulent flow field, which was generated by slightly randomizing the phases off a healthy turbulent flow and running for two full integral time scales. We checked that the different initial condition is not correlated. Then, we introduce a drop and evolve each simulation until the drop breaks. We consider that the drop has broken when we find more than one disconnected object in the binary field, β, defined as

$$\begin{aligned} \beta &= 1 \text{ if } c > 0.95 \\ \beta &= 0 \text{ if otherwise} \end{aligned} \quad (7)$$

where $c = 1$ corresponds to the phase-field inside the drop. This condition is checked every $0.1t_d$.

We have generated ensembles of simulations for We $\in$ [1.45,7.27]. The number of realizations of each ensemble is $\mathcal{N}_{\mathrm{LR}} \sim 10^3$ for the LR cases and $\mathcal{N}_{\mathrm{HR}} \sim 10^2$ for the HR cases. A summary of all simulations is presented in Table 1. From each realization in the ensemble, we store the time to breakup, t_b, and the geometry of droplet right when breakup is detected. The mass leakage inherent to the Cahn-Hilliard equation (*52*) leads to a progressive reduction of the drop diameter and, hence, to an effective reduction of the We. To keep mass losses small (here <5%, leading to less than a 3% reduction in the Weber number), HR runs were stopped at $10t_d$, and LR runs at $7t_d$. The truncated simulations are considered for the statistical estimation of the breakup rate (see section S2).

Correction (27 March 2023): Due to a production error, Qi *et al.*, Unraveling of cocatalysts photodeposited selectively on facets of BiVO4 to boost solar water splitting. *Nat. Comm.* **13**, 484 (2022), was incorrectly cited as reference 15. Reference 15 has been corrected to Qi *et al.*, Fragmentation in turbulence by small eddies. *Nat. Comm.* **13**, 469 (2022).

Supplementary Materials

This PDF file includes:
Sections S1 to S5
Figs. S1 to S5
References

REFERENCES AND NOTES

1. S. Schultz, G. Wagner, K. Urban, J. Ulrich, High-pressure homogenization as a process for emulsion formation. *Chem. Eng. Technol.* **27**, 361–368 (2004).
2. A. Håkansson, Emulsion formation by homogenization: Current understanding and future perspectives. *Annu. Rev. Food Sci. Technol.* **10**, 239–258 (2019).
3. H. Watanabe, Y. Suzuki, T. Harada, Y. Matsushita, H. Aoki, T. Miura, An experimental investigation of the breakup characteristics of secondary atomization of emulsified fuel droplet. *Energy* **35**, 806–813 (2010).
4. X. Jiang, G. A. Siamas, K. Jagus, T. G. Karayiannis, Physical modelling and advanced simulations of gas–Liquid two-phase jet flows in atomization and sprays. *Prog. Energy Combust. Sci.* **36**, 131–167 (2010).
5. G. Falkovich, A. Fouxon, M. G. Stepanov, Acceleration of rain initiation by cloud turbulence. *Nature* **419**, 151–154 (2002).
6. E. Villermaux, B. Bossa, Single-drop fragmentation determines size distribution of raindrops. *Nat. Phys.* **5**, 697–702 (2009).
7. C. Garrett, M. Li, D. Farmer, The connection between bubble size spectra and energy dissipation rates in the upper ocean. *J. Phys. Oceanogr.* **30**, 2163–2171 (2000).
8. G. B. Deane, M. D. Stokes, Scale dependence of bubble creation mechanisms in breaking waves. *Nature* **418**, 839–844 (2002).
9. W. H. R. Chan, P. L. Johnson, P. Moin, J. Urzay, The turbulent bubble break-up cascade. Part 2. Numerical simulations of breaking waves. *J. Fluid Mech.* **912**, A43 (2021).
10. A. Kolmogorov, *Doklady Akad. Nauk. USSR* **66**, 825 (1949).
11. J. Hinze, Fundamentals of the hydrodynamic mechanism of splitting in dispersion processes. *AIChE J.* **1**, 289–295 (1955).
12. N. Vankova, S. Tcholakova, N. D. Denkov, I. B. Ivanov, V. D. Vulchev, T. Danner, Emulsification in turbulent flow: 1. Mean and maximum drop diameters in inertial and viscous regimes. *J. Colloid Interface Sci.* **312**, 363–380 (2007).
13. P. Perlekar, L. Biferale, M. Sbragaglia, S. Srivastava, F. Toschi, Droplet size distribution in homogeneous isotropic turbulence. *Phys. Fluids* **24**, 065101 (2012).
14. M. E. Rosti, Z. Ge, S. S. Jain, M. S. Dodd, L. Brandt, Droplets in homogeneous shear turbulence. *J. Fluid Mech.* **876**, 962–984 (2019).

15. Y. Qi, S. Tan, N. Corbitt, C. Urbanik, A. K. R. Salibindla, R. Ni, Fragmentation in turbulence by small eddies. *Nat. Comm.* **13**, 469 (2022).
16. A. Håkansson, Towards a standard method for estimating fragmentation rates in emulsification experiments. *Processes* **9**, 2242 (2021).
17. V. Hančil, V. Rod, Break-up of a drop in a stirred tank. *Chem. Eng. Process.* **23**, 189–193 (1988).
18. C. Martínez-Bazán, J. L. Montanes, J. C. Lasheras, On the breakup of an air bubble injected into a fully developed turbulent flow. Part 1. Breakup frequency. *J. Fluid Mech.* **401**, 157–182 (1999).
19. R. Andersson, B. Andersson, On the breakup of fluid particles in turbulent flows. *AIChE J.* **52**, 2020–2030 (2006).
20. S. Maaß, M. Kraume, Determination of breakage rates using single drop experiments. *Chem. Eng. Sci.* **70**, 146–164 (2012).
21. J. Solsvik, H. A. Jakobsen, Single air bubble breakup experiments in stirred water tank. *Int. J. Chem. React. Eng.* **13**, 477–491 (2015).
22. J. Vejražka, M. Zedníková, P. Stanovskỳ, Experiments on breakup of bubbles in a turbulent flow. *AIChE J.* **64**, 740–757 (2018).
23. A. Håkansson, L. Brandt, Deformation and initial breakup morphology of viscous emulsion drops in isotropic homogeneous turbulence with relevance for emulsification devices. *Chem. Engin. Sci.* **253**, 117599 (2022).
24. A. Håkansson, On the validity of different methods to estimate breakup frequency from single drop experiments. *Chem. Eng. Sci.* **227**, 115908 (2020).
25. C. Coulaloglou, L. Tavlarides, Description of interaction processes in agitated liquid-liquid dispersions. *Chem. Eng. Sci.* **32**, 1289–1297 (1977).
26. H. Luo, H. F. Svendsen, Theoretical model for drop and bubble breakup in turbulent dispersions. *AIChE J.* **42**, 1225–1233 (1996).
27. Y. Liao, D. Lucas, A literature review of theoretical models for drop and bubble breakup in turbulent dispersions. *Chem. Eng. Sci.* **64**, 3389–3406 (2009).
28. J. Solsvik, H. A. Jakobsen, Development of fluid particle breakup and coalescence closure models for the complete energy spectrum of isotropic turbulence. *Ind. Eng. Chem. Res.* **55**, 1449–1460 (2016).
29. B. Lalanne, O. Masbernat, F. Risso, A model for drop and bubble breakup frequency based on turbulence spectra. *AIChE J.* **65**, 347–359 (2019).
30. J. I. Cardesa, A. Vela-Martín, J. Jiménez, The turbulent cascade in five dimensions. *Science* **357**, 782–784 (2017).
31. A. Vela-Martín, M. Avila, Deformation of drops by outer eddies in turbulence. *J. Fluid Mech.* **929**, A38 (2021).
32. A. Håkansson, M. Crialesi-Esposito, L. Nilsson, L. Brandt, A criterion for when an emulsion drop undergoing turbulent deformation has reached a critically deformed state. *Colloids Surf.* p. 129213 (2022).
33. B. Hof, J. Westerweel, T. M. Schneider, B. Eckhardt, Finite lifetime of turbulence in shear flows. *Nature* **443**, 59–62 (2006).
34. K. Avila, D. Moxey, A. de Lozar, M. Avila, D. Barkley, B. Hof, The onset of turbulence in pipe flow. *Science* **333**, 192–196 (2011).
35. F. Ravelet, C. Colin, F. Risso, On the dynamics and breakup of a bubble rising in a turbulent flow. *Phys. Fluids* **23**, 103301 (2011).
36. S. Perrard, A. Rivière, W. Mostert, L. Deike, Bubble deformation by a turbulent flow. *J. Fluid Mech.* **920**, A15 (2021).
37. D. Ramkrishna, *Population balances: Theory and applications to particulate systems in engineering* (Academic Press, 2000).
38. W. Chan, P. Johnson, P. Moin, The turbulent bubble break-up cascade. Part 1. Theoretical developments. *J. Fluid Mech.* **912**, A42 (2021).
39. U. Frisch, *Turbulence: The legacy of A.N. Kolmogorov* (Cambridge University Press, 2018).
40. D. Buaria, A. Pumir, E. Bodenschatz, P.-K. Yeung, Extreme velocity gradients in turbulent flows. *New J. Phys.* **21**, 043004 (2019).
41. G. E. Elsinga, T. Ishihara, J. C. Hunt, Extreme dissipation and intermittency in turbulence at very high Reynolds numbers. *Proc. R. Soc. A* **476**, 20200591 (2020).
42. N. Goldenfeld, N. Guttenberg, G. Gioia, Extreme fluctuations and the finite lifetime of the turbulent state. *Phys. Rev. E* **81**, 035304 (2010).
43. T. Nemoto, A. Alexakis, Do extreme events trigger turbulence decay? – A numerical study of turbulence decay time in pipe flows. *J. Fluid Mech.* **912**, A38 (2021).
44. S. Gomé, L. S. Tuckerman, D. Barkley, Extreme events in transitional turbulence. *Philos. Trans. R. Soc. A* **380**, 20210036 (2022).
45. E. Villermaux, B. Sixou, Y. Gagne, Intense vortical structures in grid-generated turbulence. *Phys. Fluids* **7**, 2008–2013 (1995).
46. A. La Porta, G. Voth, F. Moisy, E. Bodenschatz, Using cavitation to measure statistics of low-pressure events in large-Reynolds-number turbulence. *Phys. Fluids* **12**, 1485–1496 (2000).
47. G. Soligo, A. Roccon, A. Soldati, Breakage, coalescence and size distribution of surfactant-laden droplets in turbulent flow. *J. Fluid Mech.* **881**, 244–282 (2019).
48. G. Lemoult, L. Shi, K. Avila, S. V. Jalikop, M. Avila, B. Hof, Directed percolation phase transition to sustained turbulence in Couette flow. *Nat. Phys.* **12**, 254–258 (2016).
49. D. Jacqmin, Calculation of two-phase navier–Stokes flows using phase-field modeling. *J. Comput. Phys.* **155**, 96–127 (1999).
50. F. Magaletti, F. Picano, M. Chinappi, L. Marino, C. M. Casciola, The sharp-interface limit of the Cahn–Hilliard/Navier–Stokes model for binary fluids. *J. Fluid Mech.* **714**, 95–126 (2013).
51. G. Soligo, A. Roccon, A. Soldati, Turbulent flows with drops and bubbles: What numerical simulations can tell us—Freeman scholar lecture. *J. Fluids Engin.* **143**, 080801 (2021).
52. P. Yue, C. Zhou, J. J. Feng, Spontaneous shrinkage of drops and mass conservation in phase-field simulations. *J. Comp. Phys.* **223**, 1–9 (2007).
53. S. B. Pope, Turbulent flows (2012).
54. J. Lawless, *Statistical Models and Methods for Lifetime Data* (Wiley, second edn, 2003).
55. M. Avila, A. Willis, B. Hof, On the transient nature of localized pipe flow turbulence. *J. Fluid Mech.* **646**, 127–136 (2010).

Acknowledgments: We thank D. M. Montesdeoca for help in producing Fig. 1. **Funding:** This work was supported by Deutsche Forschungsgemeinschaft (DFG), grant number AV120/7. Computing time provided by the North-German Supercomputing Alliance (HLRN) is acknowledged. **Author contributions:** A.V.-M. and M.A. designed the research and analyzed the data. A.V.-M. developed the code and performed the simulations. **Competing interests:** The authors declare that they have no competing interests. **Data and materials availability:** All data needed to evaluate the conclusions in the paper are present in the paper and/or the Supplementary Materials. A.V.-M. and M.A. claim responsibility for all figures. The computer code, analysis scripts, and all the data are published online: https://doi.pangaea.de/10.1594/PANGAEA.951271.

Submitted 8 March 2022
Accepted 15 November 2022
Published 16 December 2022
10.1126/sciadv.abp9561
