# Supplementary material for: Memoryless drop breakup in turbulence
Source: Sci Adv. 2022 Dec 16;8(50):eabp9561. doi: 10.1126/sciadv.abp9561 (PMC9757738; doi:10.1126/sciadv.abp9561)
Supplement: Supplementary file 2 — Sections S1 to S5 Figs. S1 to S5 References [file sciadv.abp9561_sm.pdf]

CORRECTED 27 MARCH 2023; SEE FULL TEXT

Science Advances
AAAS

Supplementary Materials for

Memoryless drop breakup in turbulence

Alberto Vela-Martín *et al.*

Corresponding author: Alberto Vela-Martín, alberto.vela.martin@zarm.uni-bremen.de;
Marc Avila, marc.avila@zarm.uni-bremen.de

Sci. Adv. **8**, eabp9561 (2022)
DOI: 10.1126/sciadv.abp9561

This PDF file includes:

Sections S1 to S5
Figs. S1 to S5
References

S1 The energy spectra of isotropic turbulence

The Reynolds numbers considered in this work range from small to moderate, but display features of fully developed turbulence. The kinetic energy spectrum is

$$E(k,t) = 2\pi k^2 \langle \widehat{\boldsymbol{u}}\widehat{\boldsymbol{u}}^* \rangle_k \tag{S1}$$

where $\langle \cdot \rangle_k$ denotes the average over wavenumber shells of radius k and over time, $\widehat{\cdot}$ denotes the Fourier transform and the asterisk the complex conjugate (*53*). In Fig. S1, we show the kinetic energy spectra as a function of the wavenumber, normalised with Kolmogorov units. The highest Reynolds number, $Re_\lambda = 96$, shows an incipient inertial range with a Kolmogorov scaling $E(k) \sim k^{-5/3}$, with the wavenumber related to the diameter of the drop $k_d = 2\pi/d$ within that range (marked with horizontal lines). For the lower Reynolds number, $Re_\lambda = 58$ and 31, there is not a clear inertial-range scaling, yet the drop diameters lie above the dissipative range ($d > 20\eta$).

S2 Statistical analysis to determine the breakup rate

The breakage rates are estimated by assuming a drop survival function of the form,

$$S(t) = 1 - P = \exp[(t - t_0)/\tau_{\text{true}}], \tag{S2}$$

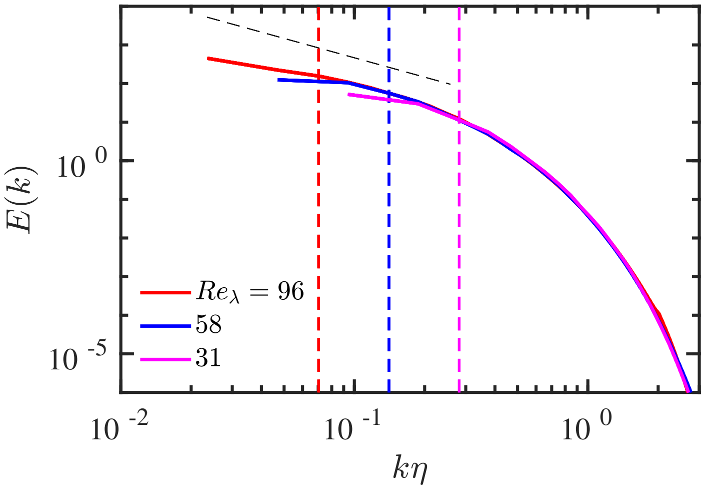

Figure S1: **Average energy spectra of isotropic turbulence at different Reynolds numbers in Kolmogorov units:** The black-dashed line mark the Kolmogorov scaling $E(k) \sim k^{-5/3}$. The horizontal colour-dashed lines mark the size of the drop in Kolmogorov units, $k_d\eta = 2\pi\eta/d$, for the three Reynolds numbers.

where $\tau_{\text{true}} = 1/\kappa_{\text{true}}$ is the characteristic lifetime of the drop (i.e. the inverse of the breakage rate). Here times are normalised with t_d. The Maximum Likelihood Estimator (MLE) of τ_{true} is the sample mean (after subtracting t_0 from the collected breakage times). As explained above, to keep mass-loss small, the runs were truncated in time and therefore the sample mean cannot be computed. Such data are known as censored data of Type-I (*54*), which requires estimating τ_{true} with a numerical method (e.g. bootstrapping).

For censored data of Type-II, in which a sample of size n is truncated after r breakups, the total run time

$$\tau = \frac{1}{r}\Big[\sum_{i=1}^{r} t_i + (n-r)t_r\Big], \tag{S3}$$

is the MLE (*54*). Here $t_0 + t_i$ is the breakage time of the ith drop and $t_0 + t_r$ the truncation time at which the simulations were truncated (*55*). The corresponding exact confidence intervals, at

level $1 - \alpha$, are

$$\tau_{\text{true}} \in \tau \times \left[2r/\chi^2_{2r,1-\alpha/2}, 2r/\chi^2_{2r,\alpha/2}\right], \tag{S4}$$

where $\chi^2_{m,p}$ is the pth quantile of the chi-squared distribution with m degrees of freedom. It is worth noting that for uncensored data ($r = n$), the sample mean is recovered as MLE, and more importantly, that the relative size of the confidence intervals is uniquely determined by the number of drops r that have broken. In the uncensored case the Central Limit Theorem yields approximate 95% confidence intervals $\tau \times (1 \pm 1.96/\sqrt{n})$, and the need for a large number of observations is a consequence of the slow convergence with n. We note that for the large sample sizes used here and also because of the additional uncertainty in determining t_0, the estimations given in (S3) and (S4) are indistinguishable from those obtained by bootstrapping (or in many cases even from the normal approximation). Hence, because of their simplicity we used equations (S3) and (S4) at 95% confidence ($\alpha = 0.05$) to estimate the values of κ and their corresponding error bars, shown in Fig 2**B**, respectively.

Figure S2**A** is a semi-logarithmic plot of the survivor function distribution at $Re_\lambda = 58$ and three selected We numbers. Their corresponding escape rates have been computed by subsequently excluding from the sample drops that have broken before t_0. Figure S2**B** shows the resulting values of $\kappa(t_0)$ as a function of t_0. Note that as t_0 is increased, the size of the sample used to estimate κ is progressively reduced from, resulting in greater error bars. In all cases the plot approaches a horizontal line for sufficiently large t_0. For $We > 2.5$, $t_0 \in [1.8, 2.6]$, whereas for $We < 2.5$, $t_0 \approx 1.75$. In order to account for the error in selecting t_0 in our analysis, the error bars shown in Fig. 2**B** are obtained using the largest and smallest $\kappa(t_0)$ in the aforementioned interval (marked with dotted lines). We found that the breakup rate depends on the Weber number only (see Figure 2), whereas t_0 was found to depend slightly on the Reynolds number and on the numerical resolution. Overall, it is very difficult to estimate t_0 precisely, especially as We increases and the time to breakup decreases.

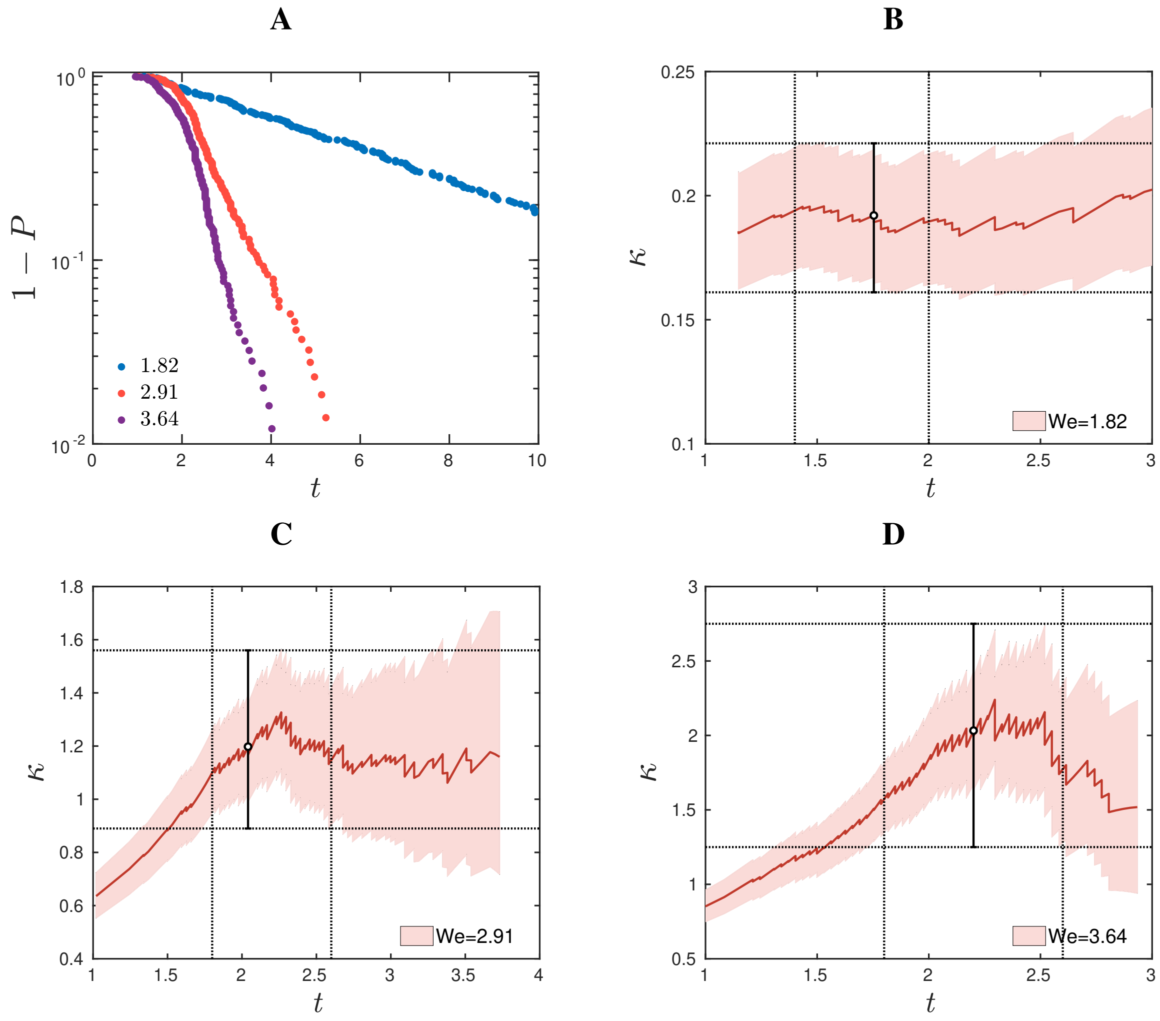

Figure S2: **Estimation of the drop breakup rate from the drop survival probability:** (**A**) Survivor function of drops for $\mathrm{Re}_\lambda = 58$, HR and We as indicated in the legend. (**B**)–(**D**) Breakage rates $\kappa(t_0) = 1/\tau(t_0)$ estimated with (S3) as a function of t_0, for We as indicated in the legend. The shaded area depicts 95% confidence intervals around κ obtained with eq. (S4). The horizontal dotted lines mark the intervals of t_0 and the vertical dotted lines the maximum and minimum $\kappa(t_0)$ in that interval, which are used as error bars in Fig. 2**B**.

S3 Breakup of ellipsoidal drops

To test the memoryless nature of breakup, we have produced simulations in which the initial drop is not spherical but ellipsoidal. In these simulations, the initial drop is an ellipsoid with principal semi-axes $A = 0.6d$ and $B = d/(2\sqrt{0.6})$, and $C = d/(2\sqrt{0.6})$. The largest axis of this ellipsoidal drop is $1.2d$, and its volume, $V = 4/3\pi ABC = 1/6\pi d^3$, is equal to the volume of the spherical drop of diameter d. Thus both drops have the same Weber number.

We have produced 200 runs with the ellipsoidal drop as the initial condition at $Re_\lambda = 58$ and $We = 2.18$, and at high-resolution (256^3 points). In Fig. S3, we show the survival probability in the ensemble with initially spherical and ellipsoidal drops. The breakup rates are statistically identical but the minimum time to breakup and the equilibration time t_0 are larger for the ellipsoidal than for the spherical drop. This shows the universal nature of the memoryless process, and indicates that t_0 is sensitive to the initial conditions. In particular, we suggest that the increase in t_0 for the ellipsoidal drop is related to the time it takes the drop to relax before it can be deformed by turbulent fluctuations.

S4 Stochastic modelling of an emulsification process

We consider a stochastic process applied to a distribution of drops with an initial mean diameter much larger than the Kolmogorov-Hinze diameter, $d_{KH} = C(\rho/\sigma)^{-3/5}\varepsilon^{-2/5}$, with $C = 0.86$, which is the value measured by Vankova *et al.* (*12*). We determine the dimensionless breakup time t_b of each drop (of Weber number We) by drawing a random number from an exponential distribution $t_{b_0} = -t_{d_0} \log X/\kappa(We)$, where $X \in [0, 1]$ is a uniform random variable, and the breakup rate is given by

$$\kappa = c_1 \exp\left(-\frac{2c_2}{We}\right), \tag{S5}$$

with $c_1 = 14.8$ and $c_2 = 3.89$ obtained from a least-square fit to our data.

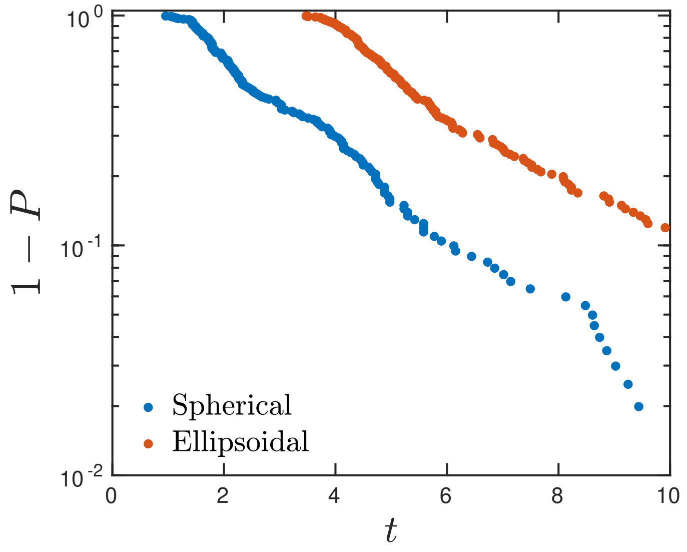

Figure S3: **Survival probability of drops which are initially spherical or ellipsoidal**: The simulations correspond to $Re_\lambda = 58$ and $We = 2.18$, and have high-resolution (256^3 points). The slope of the survival probability is the same in both cases, but the equilibration time t_0 of the ellipsoidal drop is substantially larger than that of the spherical drop.

We assume binary breakup, so that each drop breaks into two drops with total volume equal to the mother drop. The diameter distribution is drawn from a random probability distribution, which mimics the distribution observed in our direct numerical simulations. In Fig. S4**A**, we show the probability distribution of the volume of the largest daughter drop after breakup, V_1, normalised by the total volume, V_{tot}. We see that for We close to unity the distribution is centred around $V_1/V_{tot} \sim 0.75$, and that it only changes slightly with We. This is shown by the average of all distributions, which is very similar to all the distributions at different We. We model this distribution as a half-cosine distribution between for $V_1/V_{tot} \in [0.42, 1.0]$, $P(V_1/V_{tot}) = \cos(\pi/2(V_1/V_{tot} - 0.71)/0.29)$, shown as a dashed line in Fig. S4**A**.

The simulations start with a distribution of drops with a prescribed mean diameter much larger than the Kolmogorov-Hinze diameter. Initially, the distribution evolves very fast because

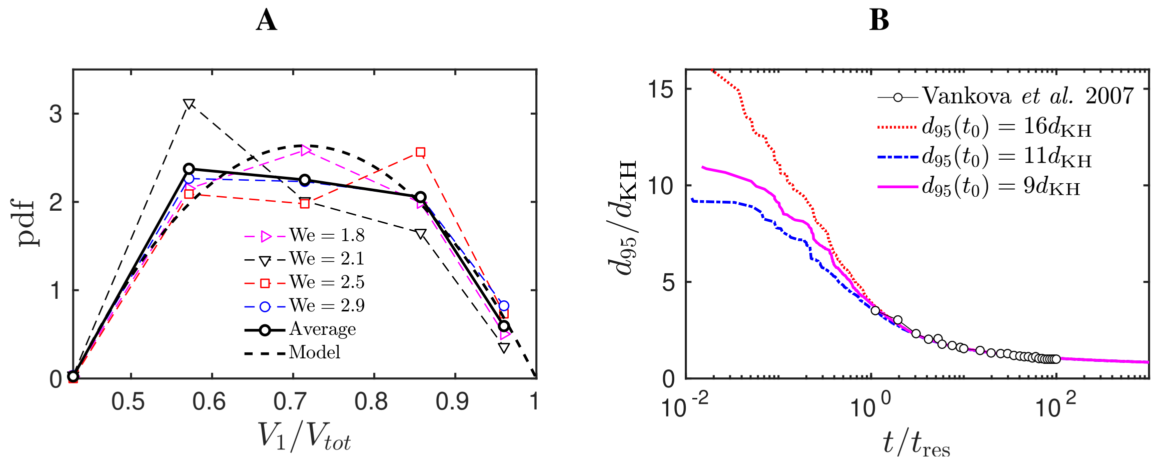

Figure S4: **Stochastic model for the evolution of dilute emulsions:** (**A**) Probability distribution of the largest daughter drop after breakup normalised by the total volume, V_1/V_{tot}, as a function of the We. The solid line denotes the average of all distributions, and the dashed line without marker the half-cosine distribution used in the stochastic breakup model. (**B**) Evolution of d_{95} in the stochastic simulation for three different initial conditions.

of the large breakup rates. Due to the stochastic nature of the breakup process, and the random multipliers applied to the volume of the daughter drops, the drop-size distribution looses the memory of the initial conditions. This is shown in Fig. S4**B**, where we plot the evolution of d_{95} for three initial conditions with different d_{95}. Note how after a time equivalent to a single passage through the emulsifier, all three initial conditions converge to the same curve, which matches the experimental measurements of Vankova *et al.* (*12*) very well thereafter.

The residence time of the drops in the turbulent regions in each passage through their emulsifier can be estimated from the physical parameters and the geometrical data as

$$t_{res} = \frac{V_{turb}}{Q}, \tag{S6}$$

where $Q = 0.145 \cdot 10^{-3}\ \mathrm{m^3 s^{-1}}$ is the volume flux and $V_{turb} = 2\pi R L^2$ is the volume of the turbulent region inside the emulsifier. Here $R = 7 \cdot 10^{-3}\ \mathrm{m}$ is the radius of the emulsification device, and $L = 4 \cdot 10^{-4}\ \mathrm{m}$ the size of the slit. Hence, $t_{res} = 3.8 \cdot 10^{-3}\mathrm{s}$. In their experiment

$d_{KH} = 9 \cdot 10^{-6}$ m and $\varepsilon = 270,000$ m^{2}s^{-3}. This value is very close to $t_{\rm res} = 2.32 \cdot 10^{-3}$s, which provides the best fit of our data to their experimental results when ε, ρ and σ are taken as in the experiments. Note that for comparing our data with their experiments, the dimensionless breakup times have to be converted to physical units by scaling it with the inertial time-scale of the drop, $t_d = (d^2/\varepsilon)^{1/3}$, which depends on the drop diameter d and hence varies at each step of the cascade breakup process. In Fig. S5**A** we show the dimensional breakup rate as a function of the drop diameter for the experiments of Vankova *et al.* (*12*).

S5 Estimates of the emulsification time scales

An analytical prediction of the temporal evolution of d_{95} is possible by considering a deterministic cascade of even binary breakups with the time to breakup given by $\tau = \kappa^{-1}$. The time (in physical time units) it takes for a drop of diameter d_0 to breakup n times to diameter $d_n = 2^{-n/3} d_0$ is

$$\tau_n = \sum_{i=0}^{n-1} \left(\frac{d_i^2}{\varepsilon}\right)^{1/3} \frac{1}{c_1} \exp\left(\frac{2c_1\sigma}{\rho\varepsilon^{2/3} d_i^{5/3}}\right), \tag{S7}$$

where $d_i = 2^{-i/3} d_0$ is the diameter at the i-th step of the breakup cascade. Using eq. (S7), and adding breakup times, we derive an analytical expression that allows to estimate the total time it takes an emulsion to reach the Kolmogorov-Hinze diameter,

$$T = \sum_{i=1}^{m} \left(\frac{d_{KH,i}^2}{\varepsilon}\right)^{1/3} \frac{1}{c_1} \exp\left(\frac{2c_1\sigma}{\rho\varepsilon^{2/3} d_{KH,i}^{5/3}}\right), \tag{S8}$$

where $d_{KH,i} = 2^{i/3} d_{KH}$, and m is such that $d_{KH,m} \sim d_0$. To evaluate this expression only the physical properties of the mixture, σ and ρ, the typical initial drop size distribution, d_0, and the kinetic energy dissipation are required.

In Fig. S5**B**, we show the characteristic breakup times used in eq. (S8) as a function of the diameter in physical units when our analytical model is applied to the experiments of Vankova

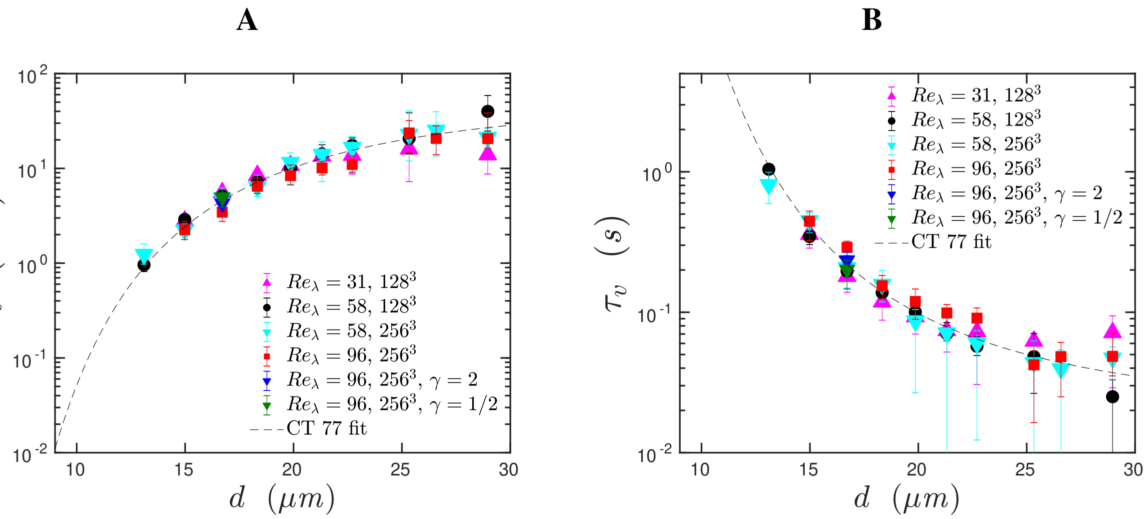

Figure S5: **Dimensional characteristic breakup times:** (**A**) Breakup rates, $\kappa_v = \kappa/t_d$, and (**B**) characteristic breakup times, $\tau_v = \tau t_d$, as a function of the diameter, d, in physical units (seconds and micrometers) corresponding to the experiment of Vankova *et al.* (*12*). The total residence time of the drops in the emulsifier after 100 passages is approximately 0.38s.

et al. (*12*). Note how, for large diameters, the breakup times are similar. This is so because, although the dimensionless breakup time increases with decreasing diameter, $c_1^{-1}\exp(2c_2\sigma/\rho\varepsilon^{2/3}d^{5/3})$, the time scale of the drop, $t_d = (d^2/\varepsilon)^{1/3}$, decreases with the diameter, leading to a plateau of the breakup times. As the diameter decreases, the exponential form of the breakup time dominates.

REFERENCES AND NOTES

1. S. Schultz, G. Wagner, K. Urban, J. Ulrich, High-pressure homogenization as a process for emulsion formation. *Chem. Eng. Technol.* **27**, 361–368 (2004).

2. A. Håkansson, Emulsion formation by homogenization: Current understanding and future perspectives. *Annu. Rev. Food Sci. Technol.* **10**, 239–258 (2019).

3. H. Watanabe, Y. Suzuki, T. Harada, Y. Matsushita, H. Aoki, T. Miura, An experimental investigation of the breakup characteristics of secondary atomization of emulsified fuel droplet. *Energy* **35**, 806–813 (2010).

4. X. Jiang, G. A. Siamas, K. Jagus, T. G. Karayiannis, Physical modelling and advanced simulations of gas–Liquid two-phase jet flows in atomization and sprays. *Prog. Energy Combust. Sci.* **36**, 131–167 (2010).

5. G. Falkovich, A. Fouxon, M. G. Stepanov, Acceleration of rain initiation by cloud turbulence. *Nature* **419**, 151–154 (2002).

6. E. Villermaux, B. Bossa, Single-drop fragmentation determines size distribution of raindrops. *Nat. Phys.* **5**, 697–702 (2009).

7. C. Garrett, M. Li, D. Farmer, The connection between bubble size spectra and energy dissipation rates in the upper ocean. *J. Phys. Oceanogr.* **30**, 2163–2171 (2000).

8. G. B. Deane, M. D. Stokes, Scale dependence of bubble creation mechanisms in breaking waves. *Nature* **418**, 839–844 (2002).

9. W. H. R. Chan, P. L. Johnson, P. Moin, J. Urzay, The turbulent bubble break-up cascade. Part 2. Numerical simulations of breaking waves. *J. Fluid Mech.* **912**, A43 (2021).

10. A. Kolmogorov, *Doklady Akad. Nauk. USSR* (1949), vol. **66**, p. 825.

11. J. Hinze, Fundamentals of the hydrodynamic mechanism of splitting in dispersion processes. *AIChE J.* **1**, 289–295 (1955).

12. N. Vankova, S. Tcholakova, N. D. Denkov, I. B. Ivanov, V. D. Vulchev, T. Danner, Emulsification in turbulent flow: 1. Mean and maximum drop diameters in inertial and viscous regimes. *J. Colloid Interface Sci.* **312**, 363–380 (2007).

13. P. Perlekar, L. Biferale, M. Sbragaglia, S. Srivastava, F. Toschi, Droplet size distribution in homogeneous isotropic turbulence. *Phys. Fluids* **24**, 065101 (2012).

14. M. E. Rosti, Z. Ge, S. S. Jain, M. S. Dodd, L. Brandt, Droplets in homogeneous shear turbulence. *J. Fluid Mech.* **876**, 962–984 (2019).

15. Y. Qi, S. Tan, N. Corbitt, C. Urbanik, A. K. R. Salibindla, R. Ni, Fragmentation in turbulence by small eddies. *Nat. Commun.* **13**, 469 (2022).

16. A. Håkansson, Towards a standard method for estimating fragmentation rates in emulsification experiments. *Processes* **9**, 2242 (2021).

17. V. Hančil, V. Rod, Break-up of a drop in a stirred tankZerfall eines tropfens in einem rührbehälter. *Chem. Eng. Process.* **23**, 189–193 (1988).

18. C. Martínez-Bazán, J. L. Montanes, J. C. Lasheras, On the breakup of an air bubble injected into a fully developed turbulent flow. Part 1. Breakup frequency. *J. Fluid Mech.* **401**, 157–182 (1999).

19. R. Andersson, B. Andersson, On the breakup of fluid particles in turbulent flows. *AIChE J.* **52**, 2020–2030 (2006).

20. S. Maaß, M. Kraume, Determination of breakage rates using single drop experiments. *Chem. Eng. Sci.* **70**, 146–164 (2012).

21. J. Solsvik, H. A. Jakobsen, Single air bubble breakup experiments in stirred water tank. *Int. J. Chem. React. Eng.* **13**, 477–491 (2015).

22. J. Vejražka, M. Zedníková, P. Stanovský, Experiments on breakup of bubbles in a turbulent flow. *AIChE J.* **64**, 740–757 (2018).

23. A. Håkansson, L. Brandt, Deformation and initial breakup morphology of viscous emulsion drops in isotropic homogeneous turbulence with relevance for emulsification devices. *Chem. Engin. Sci.* **253**, 117599 (2022).

24. A. Håkansson, On the validity of different methods to estimate breakup frequency from single drop experiments. *Chem. Eng. Sci.* **227**, 115908 (2020).

25. C. Coulaloglou, L. Tavlarides, Description of interaction processes in agitated liquid-liquid dispersions. *Chem. Eng. Sci.* **32**, 1289–1297 (1977).

26. H. Luo, H. F. Svendsen, Theoretical model for drop and bubble breakup in turbulent dispersions. *AIChE J.* **42**, 1225–1233 (1996).

27. Y. Liao, D. Lucas, A literature review of theoretical models for drop and bubble breakup in turbulent dispersions. *Chem. Eng. Sci.* **64**, 3389–3406 (2009).

28. J. Solsvik, H. A. Jakobsen, Development of fluid particle breakup and coalescence closure models for the complete energy spectrum of isotropic turbulence. *Ind. Eng. Chem. Res.* **55**, 1449–1460 (2016).

29. B. Lalanne, O. Masbernat, F. Risso, A model for drop and bubble breakup frequency based on turbulence spectra. *AIChE J.* **65**, 347–359 (2019).

30. J. I. Cardesa, A. Vela-Martín, J. Jiménez, The turbulent cascade in five dimensions. *Science* **357**, 782–784 (2017).

31. A. Vela-Martín, M. Avila, Deformation of drops by outer eddies in turbulence. *J. Fluid Mech.* **929**, A38 (2021).

32. A. Håkansson, M. Crialesi-Esposito, L. Nilsson, L. Brandt, *Colloids Surf.* p. 129213 (2022).

33. B. Hof, J. Westerweel, T. M. Schneider, B. Eckhardt, Finite lifetime of turbulence in shear flows. *Nature* **443**, 59–62 (2006).

34. K. Avila, D. Moxey, A. de Lozar, M. Avila, D. Barkley, B. Hof, The onset of turbulence in pipe flow. *Science* **333**, 192–196 (2011).

35. F. Ravelet, C. Colin, F. Risso, On the dynamics and breakup of a bubble rising in a turbulent flow. *Phys. Fluids* **23**, 103301 (2011).

36. S. Perrard, A. Rivière, W. Mostert, L. Deike, Bubble deformation by a turbulent flow. *J. Fluid Mech.* **920**, A15 (2021).

37. D. Ramkrishna, *Population balances: Theory and applications to particulate systems in engineering* (Academic Press, 2000).

38. W. Chan, P. Johnson, P. Moin, The turbulent bubble break-up cascade. Part 1. Theoretical developments. *J. Fluid Mech.* **912**, A42 (2021).

39. U. Frisch, *Turbulence: The legacy of A.N. Kolmogorov* (Cambridge University Press, 2018).

40. D. Buaria, A. Pumir, E. Bodenschatz, P.-K. Yeung, Extreme velocity gradients in turbulent flows. *New J. Phys.* **21**, 043004 (2019).

41. G. E. Elsinga, T. Ishihara, J. C. Hunt, Extreme dissipation and intermittency in turbulence at very high Reynolds numbers. *Proc. R. Soc. A* **476**, 20200591 (2020).

42. N. Goldenfeld, N. Guttenberg, G. Gioia, Extreme fluctuations and the finite lifetime of the turbulent state. *Phys. Rev. E* **81**, 035304 (2010).

43. T. Nemoto, A. Alexakis, Do extreme events trigger turbulence decay? – A numerical study of turbulence decay time in pipe flows. *J. Fluid Mech.* **912**, A38 (2021).

44. S. Gomé, L. S. Tuckerman, D. Barkley, Extreme events in transitional turbulence. *Philos. Trans. R. Soc. A* **380**, 20210036 (2022).

45. E. Villermaux, B. Sixou, Y. Gagne, Intense vortical structures in grid-generated turbulence. *Phys. Fluids* **7**, 2008–2013 (1995).

46. A. La Porta, G. Voth, F. Moisy, E. Bodenschatz, Using cavitation to measure statistics of low-pressure events in large-Reynolds-number turbulence. *Phys. Fluids* **12**, 1485–1496 (2000).

47. G. Soligo, A. Roccon, A. Soldati, Breakage, coalescence and size distribution of surfactant-laden droplets in turbulent flow. *J. Fluid Mech.* **881**, 244–282 (2019).

48. G. Lemoult, L. Shi, K. Avila, S. V. Jalikop, M. Avila, B. Hof, Directed percolation phase transition to sustained turbulence in Couette flow. *Nat. Phys.* **12**, 254–258 (2016).

49. D. Jacqmin, Calculation of two-phase navier–Stokes flows using phase-field modeling. *J. Comput. Phys.* **155**, 96–127 (1999).

50. F. Magaletti, F. Picano, M. Chinappi, L. Marino, C. M. Casciola, The sharp-interface limit of the Cahn–Hilliard/Navier–Stokes model for binary fluids. *J. Fluid Mech.* **714**, 95–126 (2013).

51. G. Soligo, A. Roccon, A. Soldati, Turbulent flows with drops and bubbles: What numerical simulations can tell us—Freeman scholar lecture. *J. Fluids Engin.* **143**, 080801 (2021).

52. P. Yue, C. Zhou, J. J. Feng, Spontaneous shrinkage of drops and mass conservation in phase-field simulations. *J. Comp. Phys.* **223**, 1–9 (2007).

53. S. B. Pope, Turbulent flows (2012).

54. J. Lawless, *Statistical Models and Methods for Lifetime Data* (Wiley, second edn, 2003).

55. M. Avila, A. Willis, B. Hof, On the transient nature of localized pipe flow turbulence. *J. Fluid Mech.* **646**, 127–136 (2010).
